# Prevalence of Delayed Initiation of Breastfeeding and Its Associated Factors Among Mothers Who Gave Birth by Cesarean Section in Gamo and Gofa Zones, Southern Ethiopia: A Multicenter Cross-Sectional Study

**DOI:** 10.1155/jnme/9554820

**Published:** 2025-04-22

**Authors:** Arega Abebe Lonsako, Tsehaynew Kasse, Aster Dure, Abera Cheru, Kinde Kibe, Addisalem Haile

**Affiliations:** ^1^College of Medicine and Health Sciences, Arba Minch University, Arba Minch, Ethiopia; ^2^College of Health and Medical Science, Haramaya University, Harar, Ethiopia

**Keywords:** caesarean section delivery, delayed initiation of breastfeeding, Ethiopia, mothers

## Abstract

**Background:** Delayed initiation of breastfeeding after birth can negatively impact maternal and newborn health, significantly increasing neonatal mortality. Due to the rising number of cesarean deliveries, the risk of delayed breastfeeding initiation is imminent. However, there is limited evidence on delayed initiation of breastfeeding among mothers who gave birth by cesarean section in Ethiopia. Thus, this study aims to assess factors associated with delayed initiation of breastfeeding among mothers who gave birth by cesarean section in public health facilities of Gamo and Gofa zones, south Ethiopia.

**Methods:** This multicenter, facility-based, cross-sectional study was conducted across five hospitals in the Gamo and Gofa zones that offer cesarean delivery services. A consecutive sampling technique, which involves selecting every eligible subject until the desired sample size is reached, was employed to include 416 mothers who underwent cesarean sections between March 20 and May 21, 2023. Data collection was performed through an interviewer-administered questionnaire and chart reviews. The collected data were entered into Epi-Data Version 4.6 and subsequently exported to the Statistical Package for Social Sciences (SPSS) Version 26 for analysis. To identify factors associated with delayed initiation of breastfeeding, a logistic regression model was fitted, with statistical significance determined at a *p* value of less than 0.05.

**Results:** The prevalence of delayed initiation of breast feeding was 53.4% with 95% CI: (48.5, 58.2). Being primiparous (AOR = 1.9, 95% CI: 1.1, 3.5), a lack of assistance from a health professional for early initiation breastfeeding (AOR = 5.1, 95% CI: 3.0, 8.6), not applying early skin-to-skin contact (AOR = 3.3, 95% CI: 1.7, 6.4), and not receiving postcesarean counseling about early initiation of breastfeeding (AOR = 2.0, 95% CI: 1.6, 3.8) were significantly associated with delayed initiation of breastfeeding.

**Conclusions:** This study found a high prevalence of delayed breastfeeding initiation among mothers who had cesarean sections, with significant factors including primiparity, a lack of professional assistance, the absence of early skin-to-skin contact, and inadequate postcesarean counseling. To address this, targeted interventions are essential, including enhancing antenatal care services, providing comprehensive breastfeeding counseling, promoting immediate skin-to-skin contact, and ensuring professional support for mothers postdelivery.

## 1. Introduction

Delayed initiation of breastfeeding is defined as starting breastfeeding more than 1 hour after birth and can have significant health consequences [1]. The World Health Organization (WHO) and United Nations International Children's Emergency Fund [2] strongly advocate for breastfeeding within the first hour of birth, emphasizing its profound benefits for both the mother and the baby [3]. For the mother, it aids uterine contractions, reduces postpartum bleeding, promotes quicker weight loss, and lowers the risk of breast cancer later in life. Similarly, for the newborn, it is crucial for strengthening the mother-child bond and supporting cognitive and immune system development [4, 5].

Delayed initiation of breastfeeding significantly increases the risk of neonatal mortality, primarily due to hypothermia and infections. A study shows that initiating breastfeeding more than an hour after birth, but within the first 24 h, increases the risk of neonatal mortality by 40%. If breastfeeding is delayed beyond 24 h, this risk increases by 80% [6, 7]. Additionally, it can also increase the likelihood of common neonatal illnesses such as upper respiratory symptoms and vomiting. These findings highlight the critical role of early breastfeeding in reducing neonatal mortality and promoting infant health [8, 9].

Globally, neonatal mortality remains a pressing issue, with 2.4 million neonates dying within the first month of life in 2020. Alarmingly, approximately 6500 neonatal deaths occurred daily, and nearly 75% of these deaths happened within the first week of life, often from preventable causes including infections and delayed breastfeeding initiation [2, 10]. In sub-Saharan Africa, the neonatal mortality rate remains the highest globally, with 27 deaths per 1000 live births recorded in 2019 with nearly one million newborns dying within 24 h. Ethiopia, one of the top ten Sub-Saharan African countries in terms of neonatal mortality, recorded a neonatal mortality rate of 30 deaths per 1000 live births in 2019 [11].

Further, the increasing rate of cesarean delivery poses a significant challenge, as it is linked to delayed breastfeeding initiation, which can negatively impact neonatal health. Addressing this issue requires improved maternal care practices and policies [12]. Globally, the cesarean delivery rate stands at 21.1% [13]. In sub-Saharan Africa, cesarean delivery rates range from 2% to 51%, with Ethiopia's rate at 29.55%, and a recent study covering public hospitals in the Gamo, Gofa, and South Omo zones of Southern Ethiopia reported a cesarean delivery rate exceeding the World Health Organization's recommended range of 10%–15% [14, 15].

The Federal Ministry of Health (FMOH) of Ethiopia has made significant efforts to promote optimal breastfeeding practices through initiatives such as the National Nutrition Program II (NNP II) and the National Guideline on Adolescent, Maternal, Infant, and Young Child Nutrition (AMIYCN) [16]. Despite these efforts, delayed initiation of breastfeeding remains a pressing public health concern, particularly among mothers who undergo cesarean sections [17]. Nationally, 26.4% of mother's experience delays in starting breastfeeding, with considerable regional disparities. This challenge is exacerbated by weak implementation of newborn care standards and structural, social, and clinical barriers, including inadequate antenatal care, unplanned pregnancies, and the rising rate of cesarean deliveries [15, 18, 19].

Moreover, most previous studies have primarily focused on the general postpartum population, overlooking mothers who gave birth by cesarean section, particularly in the study area. Understanding the context-specific determinants of delayed initiation of breastfeeding among this group is critical for addressing the issue and improving neonatal health outcomes. Therefore, this study aimed to assess the prevalence and associated factors of delayed initiation of breastfeeding among mothers who gave birth by cesarean section in Gamo and Gofa zones of the south Ethiopia region.

## 2. Methods

### 2.1. Study Setting

A multicenter, cross-sectional study was conducted from March 20 to May 21, 2023, in five selected hospitals in the Gamo and Gofa zones of Southern Ethiopia, which are part of the Southern Ethiopia region. The administrative centers, Arba Minch (Gamo Zone) and Sawla (Gofa Zone), are located 434 km and 455 km south of Addis Ababa, the capital of Ethiopia, respectively. According to population projections based on the 2007 Ethiopian census, the study area had an estimated population of 2,658,345 in 2017/18. The study was conducted in two general hospitals (Arba Minch and Sawla) and three primary hospitals (Dilfana, Chencha, and Gerese), which provide curative, preventive, and rehabilitative services to the population [20].

### 2.2. Population

All mothers who gave birth by cesarean section in public hospitals of Gamo and Gofa zones were considered as the source population, whereas all mothers who gave birth by cesarean section in selected public hospitals during the data collection period were the study population.

### 2.3. Eligibility Criteria

All mothers who gave birth by cesarean section under spinal anesthesia were included in the study. Mothers who gave birth under general anesthesia, whose newborns were admitted to the neonatal intensive care unit, and who were unable to respond due to critical illness were excluded.

### 2.4. Sample Size Determination

The required sample size was calculated using a single population proportion formula with the following assumptions: a proportion (*p*) of 50%, as no prior studies were available in this subject matter, a 95% confidence interval, a 5% margin of error, and an additional 10% to account for the potential nonresponse. The final sample size was 422.

### 2.5. Sampling Techniques and Procedures

**Figure 1:**
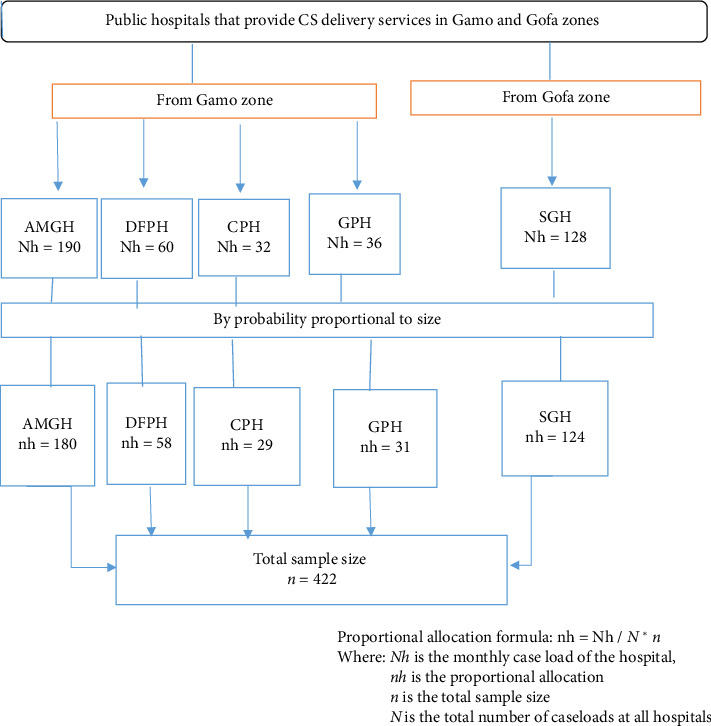
Schematic presentation of the sampling procedure used to assess factors associated with delayed initiation of breastfeeding among mothers who gave birth by cesarean section.

This study included five hospitals in the Gamo and Gofa zones that provide cesarean delivery services. The sample size was proportionally distributed based on each hospital's average monthly cesarean delivery caseload; totally, it was 446: Arba Minch General Hospital (AMGH 180), Dilfana Primary Hospital (DFPH 58), Chencha Primary Hospital (CPH 29), Gerese Primary Hospital (GPH 31), and Sawela General Hospital (SGH 124) (Figure 1).

A consecutive sampling technique was employed, recruiting women who delivered by cesarean section during the data collection period. Participants were informed about the study objectives, provided voluntary consent, and were interviewed using a structured questionnaire. No monetary or material compensation was offered, but participants were encouraged to contribute to improving maternal and child health services in the region.

### 2.6. Data Collection Instruments and Procedures

The data collection tools were adapted from WHO indicators to assess early initiation of breastfeeding [21] and modified by reviewing the relevant literature [22–26]. The tools included sections on socio-demographic characteristics, delayed initiation of breastfeeding, obstetric and health service-related factors, and newborn-related factors. The questionnaire was originally prepared in English and then translated into local languages (Gamo and Gofa). A language expert performed a back translation into English to ensure its consistency. The data were collected by six bachelor-level nurses under the supervision of four supervisors who hold master's degrees in maternity nursing. Respondents were given a brief orientation about the study's purpose and were interviewed face-to-face, with their medical record reviewed for additional information.

### 2.7. Variables of the Study

In this study, the dependent variable was delayed initiation of breastfeeding, while the explanatory variables included maternal age, educational level, employment status, place of residence, number of antenatal care visits, type of pregnancy, counseling about early initiation of breastfeeding during ANC follow-up, postnatal counseling, obstetric complications, assistance from health professionals for early initiation of breastfeeding, early skin-to-skin contact with the newborn, prior knowledge of early initiation of breastfeeding, previous breastfeeding experiences, prelacteal feeding practices, birth weight, Apgar score, and sex of the newborn.

### 2.8. Operational Definition and Measurement

#### 2.8.1. Delayed Initiation of Breastfeeding

It was defined as failure to initiate breastfeeding within 1 h after birth as WHO recommendation. The outcome variable was dichotomized as “1” for delayed initiation and “0” for early initiation [1].

#### 2.8.2. Breastfeeding

It refers to the practice of feeding an infant with milk directly from a woman's breast. Health organizations, including the WHO, recommend initiating breastfeeding within the first hour of birth and continuing as the baby desires [1].

#### 2.8.3. Prelacteal Feeding

This practice refers to providing any liquid or solid food other than the mother's breast milk to a newborn before initiating breastfeeding. Responses were recorded as “Yes” if the newborn was given any liquid or solid food other than breast milk before breastfeeding started and “No” if no such liquid or solid food was given before breastfeeding began [27].

#### 2.8.4. Health Professionals' Assistance

This refers to the support provided by healthcare professionals to help the mother and the newborn with proper attachment and positioning to start breastfeeding within the first hour after birth. If such assistance is not provided, it means that the mother did not receive professional help to initiate breastfeeding on time [28].

### 2.9. Data Quality Control

A pretest was conducted on 5% of the total sample size at the Jinka General Hospital to ensure data quality. Based on the pretest results, necessary modifications were made, and the modified tools were used for the actual data collection. The data collectors and supervisors were trained on the objectives, the data collection process, and ethical consideration. Data collectors were closely supervised and instructed to check the completeness of data.

### 2.10. Data Analysis and Processing

The collected data were cleaned, coded, and entered into the Epi-Data version 3.1 software. Then, the data were exported to SPSS version 26 for analysis. Descriptive analysis was employed to summarize the participants' characteristics, with the results presented in text, tables, and figures. A binary logistic regression model was used to explore the association between each independent variable and the outcome variable. Independent variables with a *p* value of less than 0.25 in the bivariate analysis were included in the multivariable analysis. Multicollinearity was assessed using the variance inflation factor, none of the variables yielding VIF greater than 10. While the model's fitness was evaluated using the Hosmer–Lemeshow goodness-of-fit test, it was founded to be insignificant (*p* value: 0.481). Adjusted odds ratios (AOR) with 95% confidence intervals (CI) were reported, and variables with a *p* value < 0.05 were declared to be significantly associated with delayed initiation of breastfeeding.

### 2.11. Ethical Considerations

Ethical approval was obtained from the Institutional Review Board [29] of the Arba Minch University College of Medicine and Health Sciences (IRB/1476/2023). In addition, permission was obtained from the Arba Minch General Hospital, the Dilfana Primary Hospital, the Chencha Primary Hospital, the Gerese Primary Hospital, and the Sawela General Hospital. Before data collection, informed consent was obtained from the study participants, and the right to withdraw from the interview was guaranteed. The privacy and confidentiality of the information obtained from the respondents were kept confidential and anonymous.

## 3. Results

### 3.1. Sociodemographic Characteristics of Participants

A total of 416 mothers who gave birth by cesarean section were enrolled in this study, with a response rate of 98.5%. The mean age of the participants was 28.8 years, with standard deviation (SD) of ±3.8 years. Of the respondents, 171 (41.1%) were between the ages of 25–29 years, and 229 (55%) resided in urban areas. Regarding the educational status, 244 (58.6%) had completed secondary school and above, and 214 (51.4%) mothers were housewives (Table 1).

### 3.2. Mother's Obstetric and Health Service–Related Characteristics

Among the respondents, 163 (39.2%) had fewer than four antenatal care visits, and 187 (55.0%) did not receive counseling on early breastfeeding initiation during their follow-up. 206 (49.5%) reported that they did not receive assistance from healthcare professionals to initiate breastfeeding immediately after delivery. Regarding cesarean sections, 359 (86.3%) underwent unplanned procedures. Furthermore, 225 (54.1%) did not receive postnatal counseling on early breastfeeding initiation, and 124 (29.8%) did not initiate early skin-to-skin contact. Additionally, 381 (91.5%) understood the importance of colostrum for newborns, and 286 (68.7%) practiced colostrum feeding (Table 2).

### 3.3. Newborns' Profile of Study Participants

Out of the total 416 newborns, 240 newborns (57.7%) were male. At the 1st minute after birth, 332 newborns (79.8%) had an Apgar score of seven and above, while all newborns had an Apgar score of seven and above by the 5th minute. Additionally, 385 newborns (92.5%) were born with a normal birth weight (Table 3).

### 3.4. The Prevalence of Delayed Initiation of Breastfeeding

**Figure 2:**
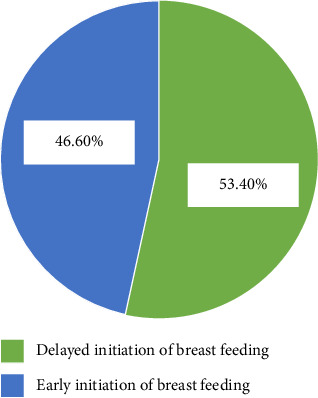
The prevalence of delayed initiation of breastfeeding among mothers who gave birth by cesarean section in this study setting.

The prevalence of delayed initiation of breastfeeding among mothers who gave birth by cesarean section in this study setting was 53.4% with 95% CI: (48.5, 58.2) (Figure 2).

### 3.5. Factors Associated With Delayed Initiation of Breastfeeding

In the binary logistic regression analysis, rural residency, being a primiparous mother, undergoing an unplanned cesarean section, a lack of assistance from a healthcare professional, not applying early skin-to-skin contact, duration of labor, not receiving postnatal counseling about early initiation of breastfeeding, and timing of delivery were factors associated with delayed initiation of breastfeeding.

However, in the multivariable logistic regression analysis, the odds of delayed initiation of breastfeeding were two times higher among primiparous mothers than multiparous mothers (AOR = 1.9, 95% CI: 1.1, 3.5). Mothers who did not receive professional support during breastfeeding initiation were five times more likely to delay breastfeeding compared to those who received support (AOR = 5.1, 95% CI: 3.0, 8.6). Additionally, the odds of delayed initiation were three times higher for mothers who did not apply early skin-to-skin contact compared to those who did (AOR = 3.3, 95% CI: 1.7, 6.4). The odds of delayed initiation of breastfeeding were two times higher among mother who did not receive postnatal counseling about early initiation of breastfeeding compared to those who did (AOR = 2.5, 95% CI: 1.6, 3.8) (Table 4).

## 4. Discussion

This study aimed to determine the prevalence of delayed initiation of breastfeeding and its associated factors among mothers who gave birth via cesarean section in public health facilities in the Gamo and Gofa zones of southern Ethiopia. The findings revealed that the prevalence of delayed initiation of breastfeeding was 53.4% (95% CI: 48.5, 58.2). Factors significantly associated with delayed initiation included being a primiparous mother, a lack of healthcare professional assistance, the absence of early skin-to-skin contact, and not receiving postnatal counseling on early breastfeeding initiation.

This finding was consistent with the results of study performed in South Sudan (52%) and Addis Ababa, Ethiopia (48.8%) [30, 31]. However, this finding was higher than the results of studies conducted in Uganda (48.2%), Gondar (48.1%), and Dessie, Ethiopia (43%) [10, 22, 23]. This discrepancy may be due to differences in eligibility criteria, healthcare practices, study settings, and sampling procedures, as well as variations in the timing of the studies.

In this study, the odds of delayed initiation of breastfeeding were two times higher among primiparous mothers than multiparous mothers. This finding was supported by the result of the Ethiopian Demographic and Health Survey in 2016 [15]. This might be because primiparous mothers often lack breastfeeding experience and confidence, requiring additional support and guidance to navigate breastfeeding challenges. Conversely, multiparous women typically possess greater confidence and skills from prior experiences, enabling them to overcome common breastfeeding obstacles more effectively [32].

Mothers who did not receive professional support during breastfeeding initiation were five times more likely to delay breastfeeding compared to those who received support. This finding is consistent with studies conducted in Gondar, Addis Ababa, Ethiopia, and Uganda [31, 33]. This may be due to insufficient professional support, particularly in proper attachment and positioning, which can hinder a mother's ability to establish successful breastfeeding practices. Hands-on assistance is crucial to guide mothers on effective breastfeeding techniques, responsive feeding, and comforting their infants [34].

Additionally, the odds of delayed initiation were three times higher for mothers who did not apply early skin-to-skin contact compared to those who did. This result is consistent with studies from Malaysia and Nepal [35, 36]. A possible justification could be that early skin-to-skin contact is essential for initiating breastfeeding immediately, as it promotes bonding, triggers breastfeeding reflexes in both the mother and the newborn, and boosts the mother's confidence. Without early practice of skin-to-skin contact, mothers may feel anxious, leading to delays in breastfeeding initiation [37, 38].

The odds of delayed initiation of breastfeeding were two times higher among mother who did not receive postnatal counseling about early initiation of breastfeeding compared to those who did. This finding aligns with a study conducted in Dessie, Ethiopia [23]. This may be because postnatal counseling is essential as it provides mothers with crucial support and information, aiding in the earlier initiation of breastfeeding. It helps to overcome potential knowledge and skill deficits related to breastfeeding that may arise after delivery. Proper counseling from healthcare providers significantly enhances the likelihood of successful breastfeeding [39].

Finally, delayed initiation of breastfeeding among mothers who gave birth by cesarean section remains a significant concern. Healthcare providers must address the unique challenges faced by these mothers, offering targeted assistance in breastfeeding positioning and attachment. A tailored approach, including specific counseling strategies, is essential to meet their needs and ensure successful breastfeeding initiation.

## 5. Conclusion

This study reveals a high prevalence of delayed initiation of breastfeeding among mothers who underwent cesarean sections, with contributing factors including primiparity, a lack of professional support, the absence of early skin-to-skin contact, and insufficient postnatal counseling. To address these issues, we recommend that health policymakers implement supportive policies ensuring all mothers, particularly first-time ones, receive comprehensive breastfeeding support during antenatal and postnatal care. Integrating immediate skin-to-skin contact into standard postcesarean protocols and enhancing the roles of community health workers through targeted training and resources are also crucial. Healthcare professionals should provide thorough counseling on breastfeeding benefits and techniques, offer continuous professional support, and promote kangaroo care practices to improve breastfeeding outcomes. Educators and researchers are encouraged to develop specialized training programs for healthcare providers and conduct further research to identify and address additional barriers to early breastfeeding initiation postcesarean.

### 5.1. Limitation of the Study

The cross-sectional design prevents establishing causal relationships between delayed initiation of breastfeeding and associated factors, as data were collected at a single point in time. Future longitudinal studies are recommended to address this. Recall bias may also have affected the accuracy of self-reported data, especially on breastfeeding initiation timing. To reduce this, we used structured questions and collected data shortly after delivery.

## Figures and Tables

**Table 1:** Sociodemographic characteristics of study participants (*n* = 416).

Variable		Frequency (*n*)	Percent (%)
Mothers' age	Less than 19 years	7	1.6
	20 to 24 years	55	13.2
	25 to 29 years	171	41.1
	30 to 34 years	160	38.4
	35 and more years	23	5.7

Educational level	No formal education	18	4.3
	Primary education	154	37
	Secondary education	157	37.8
	College and above	87	20.9

Employment status	Housewife	214	51.4
	Government employee	89	21.4
	Merchant	110	26.4
	NGO employee	3	0.7

Place of residence	Rural	187	45.0
	Urban	229	55.0

Husband's educational level	No formal education	10	2.4
	Primary education	96	23.1
	Secondary education	90	21.6
	College and above	220	52.9

**Table 2:** Obstetric and health service–related characteristics of study participants (*n* = 416).

Variables		Frequency (*n*)	Percent (%)
Parity	Primiparous	150	36.1
	Multiparous	266	63.9

Frequency of antenatal care follow-up	< 4 visits	163	39.2
	≥ 4 visits	253	60.8

Place of antenatal care follow-up	Hospital	232	55.8
	Health center	184	44.2

Counseling about early initiation of breastfeeding during antenatal care follow-up	Yes	229	45.0
	No	187	55.0

Time of labor	≤ 12 h	224	53.8
	> 12 h	192	46.2

Cesarean section type	Unplanned	359	86.3
	Planned	57	13.7

Time of the delivery	Day	213	51.2
	Night	203	48.8

Postnatal counseling about early initiation of breastfeeding	Yes	191	45.9
	No	225	54.1

Time that early skin-to-skin contact started	Within 1 h of birth	292	70.2
	After 1 h of birth	124	29.8

Understood the importance of colostrum	Yes	381	91.6
	No	35	8.4

Received health professional's assistance to initiate breastfeeding.	Yes	210	50.5
	No	206	49.5

Pregnancy-related complication	Yes	39	9.4
	No	377	90.6

Breastfeeding experience	Yes	265	63.7
	No	151	36.3

*Note:* Primiparous = having one viable pregnancy; multiparous = having more than one viable pregnancy.

**Table 3:** Newborns' profile of study participants (*n* = 416).

Variables		Frequency (*n*)	Percent (%)
Sex of the newborn	Male	240	57.7
	Female	176	42.3

Apgar score at 1st minute	Less than seven	84	20.2
	Seven and above	332	79.8

Birth weight	Normal (2.5 to 4 kg)	385	92.5
	Macrosomia (> 4 kg)	31	7.5

**Table 4:** Factors associated with delayed initiation of breastfeeding among mothers who gave birth by cesarean section in this study setting (*n* = 416).

Variables		Delayed initiation of BF		COR (95% CI)	AOR (95% CI)	*p* value
		Yes (%)	No (%)			
Residence	Urban	101 (44.1)	128 (55.9)	1	1	
	Rural	121 (64.7)	66 (35.3)	2.3 (1.5–3.4)	1.4 (0.8–2.2)	0.17

Parity	Primiparous	104 (69.3)	46 (30.7)	2.8 (1.8–4.3)	1.9 (1.1–3.5)^∗^	0.02
	Multiparous	118 (44.4)	148 (55.6)	1	1	

Cesarean section type	Planned	18 (31.6)	39 (68.4)	1	1	
	Unplanned	204 (56.8)	155 (43.2)	2.8 (1.5–5.2)	0.5 (0.2–1.2)	0.16

Health professional assistant	Yes	60 (28.6)	150 (71.4)	1	1	
	No	162 (78.6)	44 (21.4)	9.2 (5.8–14.4)	5.1 (3.0–8.6)^∗^	< 0.01

Time of the delivery	Day	104 (48.8)	109 (51.2)	1	1	
	Night	118 (58.1)	85 (41.9)	1.4 (0.9–2.1)	0.8 (0.5–1.4)	0.57

Time of skin-to-skin contact	Within 1 h	115 (39.4)	177 (60.6)	1	1	
	After 1 h	107 (86.3)	17 (13.7)	9.6 (5.5–17.0)	3.3 (1.7–6.4)^∗^	< 0.01

Time of labor	≤ 12 h	100 (44.6)	124 (55.4)	1	1	
	> 12 h	122 (63.5)	70 (36.5)	2.1 (1.4–3.2)	1.3 (0.7–2.4)	0.243

Postnatal counseling about early initiation of BF	Yes	76 (39.8)	115 (60.2)	1	1	
	No	146 (64.9)	79 (35.1)	2.7 (1.8–4.1)	2.0 (1.2–3.3)^∗^	0.01

Abbreviations: AOR = adjusted odds ratio; BF = breastfeeding; CI = confidence interval; COR = crude odds ratio.

^∗^shows significance.

## Data Availability

The data sets used and/or analyzed during the current study are available from the corresponding author upon reasonable request.

## Nomenclature

ANC: Antenatal care
AOR: Adjusted odds ratio
CI: Confidence interval
DHS: Demographic Health Survey
EDHS: Ethiopian Demographic Health Survey
SPSS: Statistical Package for Social Science
WHO: World Health Organization.
